# The association between maximal muscle strength, disease severity and psychopharmacotherapy among young to middle-aged inpatients with affective disorders – a prospective pilot study

**DOI:** 10.1186/s12888-024-05849-2

**Published:** 2024-05-29

**Authors:** Hannah Ramming, Linda Theuerkauf, Olaf Hoos, Katharina Lichter, Sarah Kittel-Schneider

**Affiliations:** 1 Department of Psychiatry, Psychosomatics and Psychotherapy, Center of Mental Health, University Hospital Würzburg, Margarete-Höppel-Platz 1, 97080 Würzburg, Germany; 2 Center for Sports and Physical Education, University of Würzburg, Am Hubland, 97074 Würzburg, Germany; 3 Department of Psychiatry and Neurobehavioural Science, Acute Mental Health Unit, University College Cork, Cork University Hospital, Wilton, Cork, T12DC4A Ireland; 4 Present Address: Institute of Science and Technology Austria, Am Campus 1, Klosterneuburg, 3400 Austria

**Keywords:** Affective disorder, Depression, Bipolar disorder, Muscle strength, Psychopharmacotherapy, Young, Physical activity

## Abstract

**Background:**

Motor alterations and lowered physical activity are common in affective disorders. Previous research has indicated a link between depressive symptoms and declining muscle strength primarily focusing on the elderly but not younger individuals. Thus, we aimed to evaluate the relationship between mood and muscle strength in a sample of *N* = 73 young to middle-aged hospitalized patients (18–49 years, mean age 30.7 years) diagnosed with major depressive, bipolar and schizoaffective disorder, with a focus on moderating effects of psychopharmacotherapy. The study was carried out as a prospective observational study at a German psychiatric university hospital between September 2021 and March 2022.

**Methods:**

Employing a standardized strength circuit consisting of computerized strength training devices, we measured the maximal muscle strength (F_max_) using three repetitions maximum across four muscle regions (abdomen, arm, back, leg) at three time points (t_1_-t_3_) over four weeks accompanied by psychometric testing (MADRS, BPRS, YRMS) and blood lipid profiling in a clinical setting. For analysis of psychopharmacotherapy, medication was split into activating (AM) and inhibiting (IM) medication and dosages were normalized by the respective WHO defined daily dose.

**Results:**

While we observed a significant decrease of the MADRS score and increase of the relative total F_max_ (rTF_max_) in the first two weeks (t_1_-t_2_) but not later (both *p* < .001), we did not reveal a significant bivariate correlation between disease severity (MADRS) and muscle strength (rTF_max_) at any of the timepoints. Individuals with longer disease history displayed reduced rTF_max_ (*p* = .048). IM was significantly associated with decreased rTF_max_ (*p* = .032). Regression models provide a more substantial effect of gender, age, and IM on muscle strength than the depressive episode itself (*p* < .001).

**Conclusions:**

The results of the study indicate that disease severity and muscle strength are not associated in young to middle-aged inpatients with affective disorders using a strength circuit as observational measurement. Future research will be needed to differentiate the effect of medication, gender, and age on muscle strength and to develop interventions for prevention of muscle weakness, especially in younger patients with chronic affective illnesses.

**Supplementary Information:**

The online version contains supplementary material available at 10.1186/s12888-024-05849-2.

## Background

Affective disorders, like major depression (MDD) and bipolar affective disorders (BD), have a median onset in young adulthood [1]. Beyond the symptoms of the mood disorder itself, affected individuals are impacted by additional burden throughout their life span, e.g., due to an increased risk for premature mortality, conveyed for example by cardiometabolic diseases. Furthermore, they suffer from lowered life quality and show reduced physical activity (PA) [2–6]. The latter might result, besides other symptoms, from (psycho)motor alterations in depressed patients which include e.g. deficits in the balance system and body’s posture or lowered muscle strength [7–10]. Secondly, psychopharmacotherapy in affective disorders and its adverse effects are likely to be influencing levels of PA and muscle strength [11, 12], which is defined as critical motor capacity or capability that underpins motor performances [13]. Lately, meta-analyses have revealed PA as increasingly important not only in the view of diagnostics but also as a therapeutic strategy for mental disorders [14, 15].

Beside reduced PA and psychopharmacotherapy, several mechanisms may contribute to a decrease in muscle strength in depressive patients. Both sarcopenia and malnutrition, especially in the elderly, are associated with reduced muscular strength [16, 17]. Molecularly, a dysregulation of the hypothalamic-pituitary-adrenal axis need to be considered as well as inflammatory processes affecting motivation and motor function by cytokine effects on dopaminergic pathways [18–20]. Further, an abnormal balance in modulation of motor networks by neurotransmitters, by non-motor networks systems, such as sensory networks, or of general cortical activity appear crucial for psychomotor alterations in depression [12, 21]. Previous studies, of which most were conducted in outpatient settings [22], primarily focused on the association between both incidence of depression and depressive symptoms and lowered muscle strength in elderly patients (for review see [23, 24]). Recent research confirmed comparable results and the positive effect of PA in inpatient settings [25–28], thereby also critically addressing the specific context of PAs for mentally ill inpatients based on e.g. physical health disparities or socioecological complexities [29, 30].

**Fig. 1:**
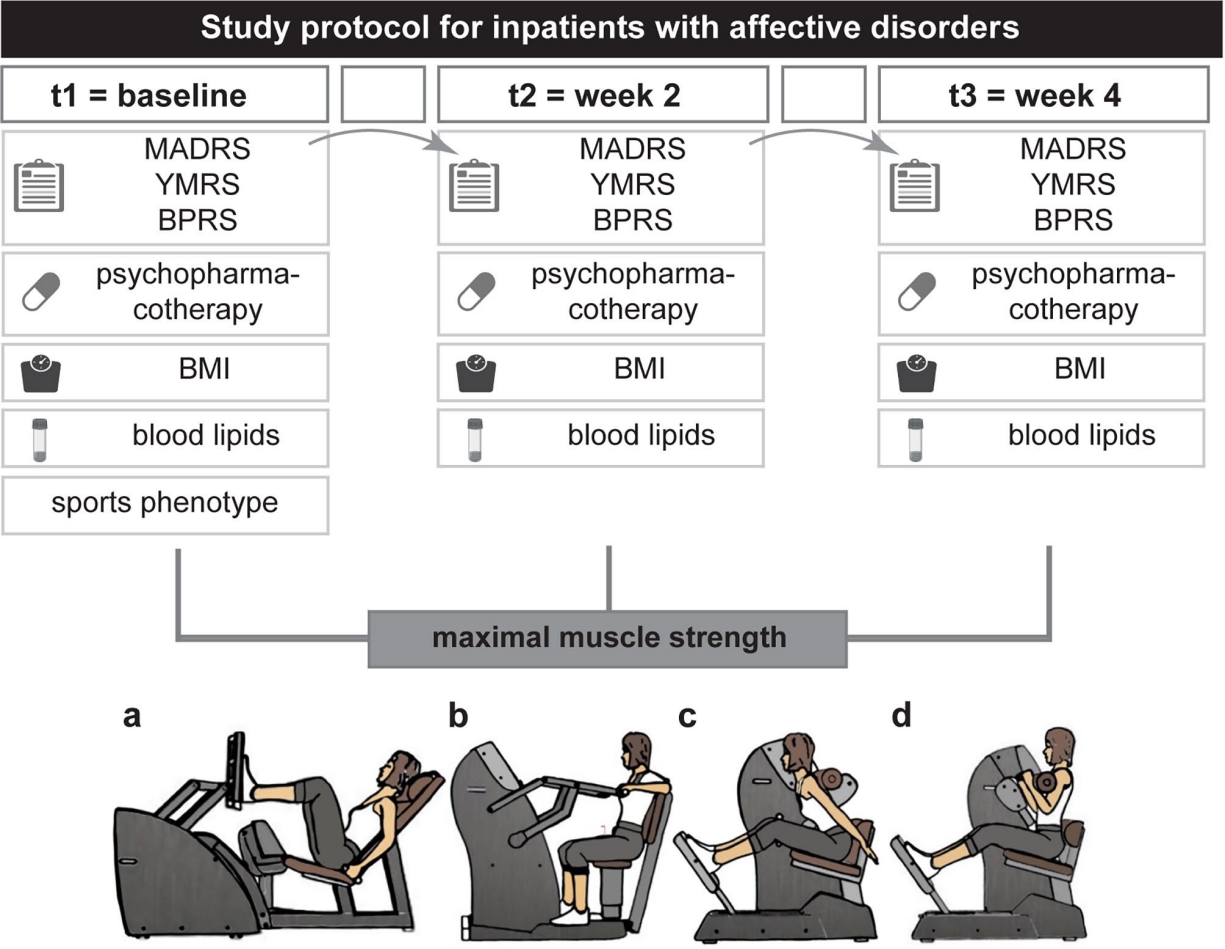
Study design. After study enrolment, patients were scheduled for standardized strength assessment at three time points (t1-t3) to measure the maximal muscle strength. The parkour consisted of consisting of four commercial fitness devices addressing leg extensors and flexors **(a)**, arm extensors and flexors **(b)**, back extensors **(c)**, and abdominal flexors **(d)**. At t1-t3, disease severity, daily medication, body weight, and selected blood parameters were assessed. *Abbreviations BPRS* Brief Psychiatric Rating Scale, *MADRS* Montgomery–Åsberg Depression Rating Scale, *t* time point *YMRS* Young Mania Rating Scale. The scheme of the fitness devices was provided by courtesy of milon industries GmbH, icons were obtained from Biorender

To our knowledge no study to date has attempted to establish a link between disease severity in young to middle-aged patients suffering from an affective disorder, muscle strength and psychopharmacotherapy in an inpatient setting. We hypothesize that severity of depressive symptoms might influence muscular strength moderated by medication effects. Thus, we aimed to evaluate this relationship in a prospective observational pilot study using a patient sample of young to middle-aged inpatients diagnosed with MDD, BD and schizoaffective disorder (SZD). Applying naturalistic psychopharmacotherapy, we monitored the inpatients’ maximal muscle strength at three time points over four weeks with a computerized strength circuit accompanied by psychometric testing for depression severity and disease parameters, blood diagnostics, and evaluation of inhibiting and activating medication effects (Fig. 1).

## Methods

### Study sample description

**Fig. 2:**
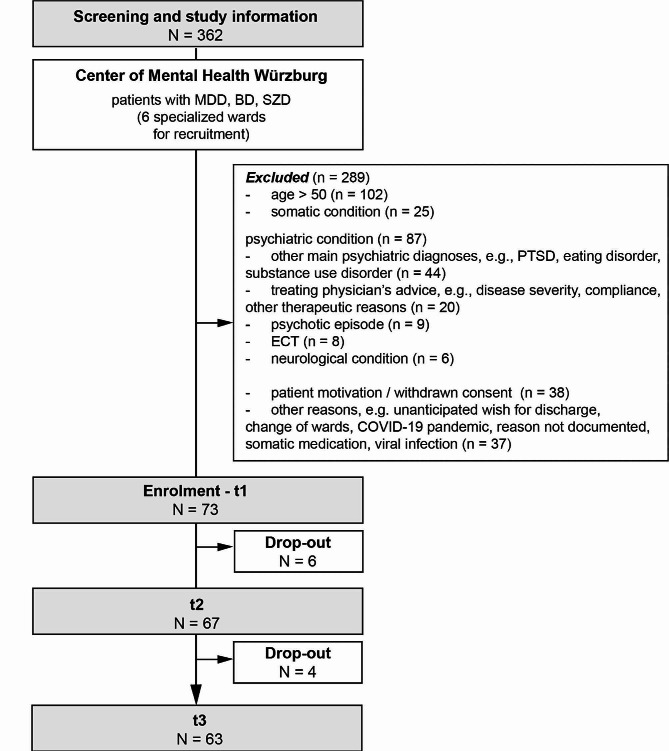
Consort chart. Overview of screening and enrolment process resulting in a final study sample of *N* = 73 participants at baseline. Abbreviation: *BD* bipolar disorder, *ECT* electro-convulsive therapy, *MDD* major depressive disorder, *N/n* sample number, *PTSD* post-traumatic stress disorder, *SZD* schizo-affective disorder

The study was carried out as a prospective observational study of mentally ill patients between September 2021 and March 2022 with three time points of measurement (t_1_ = enrolment, t_2_ = week 2, t_3_ = week 4) and a total duration of four weeks (Fig. 1). Participants were recruited by specialized staff within the inpatient setting of the Department of Psychiatry, Psychotherapy and Psychosomatic Medicine, University Hospital Würzburg, Germany (Fig. 2). Mentally ill inpatients aged 18 years to 50 years suffering from either [1] an affective disorder (Statistical Classification of Diseases and Related Health Problems Version 10 (ICD-10) F30-F39) or [2] schizoaffective disorder (ICD-10 F25) diagnosed by a consultant were eligible. A further inclusion criterion were sufficient German language skills. Potentially eligible patients were examined within the first two weeks of their inpatient stay with regards to their ability to give informed consent to the study participation by the treating psychiatrists and the study psychiatrist (SKS). Only patients who had the capacity to give informed consent were included. We did not include patients who would have required a legal guardian. This procedure was approved by the ethics committee of the University Hospital Würzburg (approval no. 35/21). Exclusion criteria were defined as (i) inability to give written informed consent, (ii) severe neurological condition (incl. neuromuscular diseases, epilepsy or stroke ≤ three months ago), (iii) recent orthopaedic surgery, (iv) cardiovascular diseases (insufficiently controlled hypertonus, severe heart insufficiency (> New York Heart Association class 1) or heart attack ≤ three months ago), (v) severe kidney insufficiency, (vi) insufficiently controlled diabetes mellitus, (vii) pregnancy and lactation. Psychiatric treatment was carried out naturalistically according to the treating physician’s choice, thus, no pre-established length of in-patient stay was defined.

In total *N* = 362 inpatients were initially screened of which *N* = 289 patients had to be excluded (Fig. 2). In total, *N* = 73 patients were enrolled in our study protocol and underwent measurements at t_1_. Due to unanticipated wishes for discharge of *N* = 6 patients and of *N* = 4 patients before t_2_ and t_3_, respectively, *N* = 63 patients completed the entire study protocol. The procedures were approved by the local ethics committee of the University Hospital of Würzburg (approval no. 35/21) and were carried out in accordance with the ethical standards of the Declaration of Helsinki [31].

### Primary outcome parameters and study endpoint

**Fig. 3:**
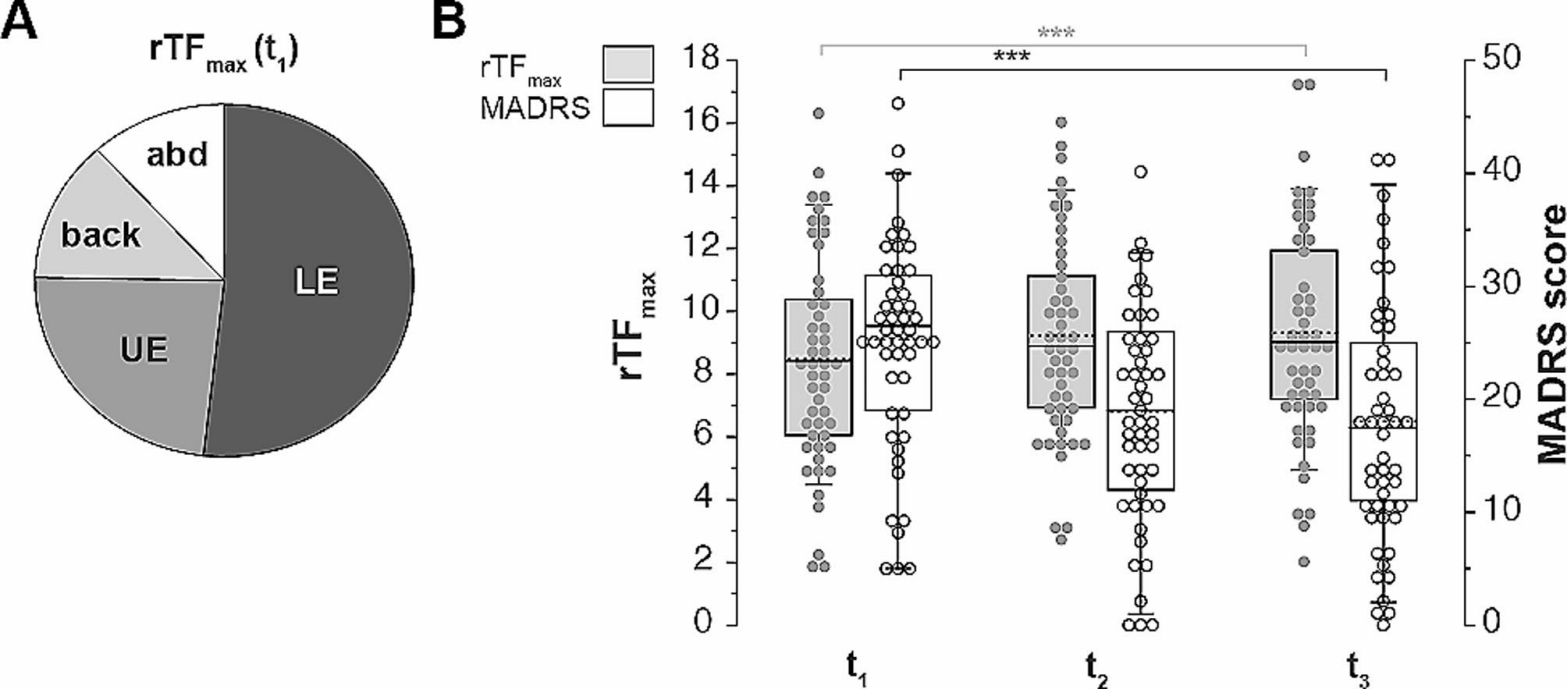
The development of maximal muscle strength and disease severity over the study course.** A**: Percentual distribution of the relative total muscle strength (rTF_max_) comprising lower extremities (LE), upper extremities (UE), back and abdomen (abd), at time point 1 (t_1_, *N* = 70). **B**: Summary graphs of rTF_max_ (light grey) and the MADRS (white) scores over the study course (*N* = 50, p = < 0.001 after Tukey and Bonferroni correction, Friedman test (rTF_max_), repeated measures ANOVA (MADRS)). Horizontal lines in boxplots represent median, dotted horizontal lines represent mean; boxes 25th and 75th quartiles; whiskers 5th and 95th percentiles; scatter plots show individual data points. Asterisks indicate significance level (*p < .05, **p < .01, ***p < .001)

Primary outcome parameters of the study were defined as the maximal muscle strength (F_max_) and depression severity (Montgomery Ǻsberg Depression Rating Scale, MADRS, Fig. 3). Due to the low sample number (*N* = 2 (2.7%) participants with manic and *N* = 9 (8.2%) participants with mixed episodes), manic and mixed episodes in BD were not investigated and data setsexcluded from the correlation calculation of depression severity and F_max_. To account for interindividual differences, F_max_ and MADRS scores were compared at the time points t_1_-t_3_. For intraindividual differences, score deltas between timepoints were calculated and compared. The study endpoint was defined at t_3_ after four weeks. Data of psychopharmacotherapy, other psychometric parameters, somatic and laboratory biomarkers were secondary outcome parameters and used for evaluation of and/or correlation with F_max_ and the MADRS score in the whole sample.

### Minimal recruitment number

For the study protocol, the minimum recruitment number of participants was defined by a power analysis for the expected differences of F_max_ and MADRS scores defined as the primary outcome parameters. We calculated a minimum recruitment number of *N* = 53 participants and assumed, based on existing studies and a meta-analysis for older patients as well as clinical observations [32], a moderate effect in our study cohort (Pearson’s *r* = .3).

### Psychometric testing

Psychometric evaluation of patients was carried out in scheduled study interview rounds at t_1_-t_3_. Disease severity and dimension were assessed by the MADRS [33]. Therapy response was defined as a ≥ 50% reduction (t_3_) of the baseline MADRS (t_1_); remission was defined as a MADRS score ≤ 12 at t_3_ [34].

The Brief Psychiatric Rating Scale (BPRS [35]), was used as a screening tool for psychotic symptoms. Baseline severity of the current manic episode was evaluated by the Young Mania Rating Scale (YMRS [36]). At t_1_, the following questionnaires were used to assess possible substance use disorders: Fägerstrom questionnaire [37] evaluating nicotine addiction and the Alcohol Use Disorders Identification Test (AUDIT [38], evaluating alcohol consumption. A suspected alcohol related disorder was defined as an AUDIT score ≥ 8. To assess chronic stress levels and life quality, patients were evaluated with the Trier Inventory for Chronic Stress (TICS [39]), and the Short-Form-36 Health Survey (SF-36 [40]).

### Strength testing procedure

For a standardized measurement of F_max_ in the selected muscle regions, patients were introduced to a strength circuit consisting of computerized strength training devices (milon industries GmbH, Emersacker, Germany) of the following type: miltronic PREMIUM med “Leg Press”, “Chest Press”, “Back Extension”, “Abdominal Crunch”. The strength training device guided the execution of the individual movement; prior knowledge of the participant was not required. General testing conditions included the presence of only the investigator and the patient for measurements. Further, patients underwent the measurement which was conducted by two investigators for the entire cohort during daytime (9am-5pm). Due to the complexity of the individual therapeutical program for mentally ill inpatients and the availability of the training devices only in another hospital department, the exact time could not be standardized. At the computerized strength training devices, the maximal dynamic force of the muscle group was quantified in the isokinetic (dynamometry) modus under standardized and reliable testing conditions, especially under constant angle velocity [41, 42]. The measurement technique of three repetitions maximum was based on the principle of repetition maximum zones [13, 43–45], which were previously used with good reliability at this specific device type [46–48]. According to the manufacturer milon industries GmbH, the accuracy of the computerized measurement was specified with ± 10%. The strength circuit was not designed as a training intervention for the patients. Due to the number of measurements and the study duration of four weeks, we assumed that neither the measurements nor personal training had a significant effect on muscle strength and muscle mass [49–51].

At t_1_-t_3_, patients underwent the following measurement procedure:

i) Based on the patient’s height and weight, a biometric recognition software (MILONIZER 1, version 1.4.1.0, milon industries GmbH, Emersacker, Germany) automatically adjusted the machine to the individual user. In ca. 10% of the cases, manual adjustments had to be performed if the patient’s body configuration, especially a very short body length in female patients, did not match to the automatically adjusted positions of the training devices. The criterium for adjustment which was carried out under supervision of the physiotherapists was the inability to accurately perform the entire movement.
ii) General warm-up using a cross trainer (device type “Crosswalker”, milon industries GmbH, Emersacker, Germany) at 25 W for 10 min.
iii) Specific warm-up consisting of a brief training (1 min) and break (30–45 s) per device [48]. For the concentric part of the movement, weight resistance was adjusted to 25 kg at the device “Leg Press”. For the other three devices, weight resistance was accustomed to 12 kg. For the eccentric part of the movement, the weight resistance was raised to 110% of the initial weight resistance (standard device settings).
iv) Measurement of the three repetitions maximum for the dynamic F_max_ at each device were carried out by verbally instructing the patient to achieve the maximal performance. Each of the three measurements was separated by a break of 10 s. The patient completed the strength circuit in a specified order of the devices as listed above, separated by a break of 1 min between each device. Absolute values of dynamic F_max_ (unit: kg) for each muscle group were automatically calculated by the device’s software CARE (milon industries GmbH, Emersacker, Germany). The highest value out of three measurements was used, as some patients had difficulties to execute the guided movement properly. For calculation, F_max_ values were normalized by division by the patient’s BMI [52–54] and summated to a relative total F_max_ (rTF_max_).

### Psychopharmacotherapy

At t_1_-t_3,_ daily psychopharmaceutical and other medication were documented. Substances were included if participants received them within 24 h prior to the measurement. As an approximation model, we differentiated the drugs in two subgroups, activating (AM) and inhibiting (IM) medication based on [55], detailed in Supplementary Table 1. The defined daily dose (DDD), which is the assumed average maintenance dose per day for a drug used for its main indication in adults (WHO; [56]), was introduced as normalizing parameter [57]. Thus, a relative dose (rD) was determined for each medication. When multiple medications were taken simultaneously, the individual values of rD for the activating (rD_AM_) and inhibiting (rD_IM_) medication groups were added. The described method assumes that the number of effective molecules is directly proportional to the administered dosage. The difference between rD_AM_ and rD_IM_ was termed as total relative dosage (rTD) for which a positive (AM) and negative (IM) algebraic sign was to indicate more activating (+) or inhibiting (-) change of medication over time.

### Therapeutical sports program

For each patient, participation in the therapeutical sports program of the hospital offered by physiotherapists was documented between t_1_-t_2_ and t_2_-t_3_. It comprised activity groups of different intensity and duration (0.5–1 h), such as the morning sport, Nordic walking, afternoon activity, and the fitness group. For a detailed description of the sports groups see Supplementary Table 2. Participation in the therapeutical sports program was highly recommended by the therapeutic team but not mandatory. The course of the sports therapy session was adjusted depending on the mental and physical condition of the participants. One of the main intentions of the sports therapy group is a supervised PA within a stationary inpatient setting with reduced possibility for daily activities and movement. Regularly, every inpatient was assigned to two or three sports groups based on group availability and individual training status. To differentiate between intensities, sports groups were classified with a one-dimensional intensity factor of either 0.5 (low) or 1 (high). The product of the intensity factor and the duration of the individual sports unit were summated between t_1_-t_2_ and t_2_-t_3_ and used for further calculations. In addition, duration and frequency of sports activities before admission, defined as a leisure-time physical activities which were planned, structured, repetitive, and purposefully conducted to improve or maintain one or more components of physical fitness as an objective [58, 59], were documented in the study admission process.

### Further procedures

Patients were scheduled for blood sampling to perform routine blood analysis of the complete blood count, total cholesterol (TC), high density lipoprotein (HDL), low density lipoprotein (LDL), creatine kinase (CK) and triglycerides (TG) at all three time points. Blood sample collection was carried out by trained staff.

### Statistics

All statistical calculations were carried out with the IBM SPSS software (Version: 26 and 28, IBM) and Sigma Plot 14 (Systat Software GmbH). Asterisks in figures and tables indicate the level of significance (∗*p* < .05, ∗∗*p* < .01, ∗∗∗*p* < .001), n/N is used as abbreviation for the sample number. For all data, normality was tested using Shapiro-Wilk tests; parametric data are reported as a mean ± SD. Non-parametric are reported as a median (25th -75th percentile). Correlations were calculated using the Pearson correlation coefficient based on the assumption of linearity. Data assessed at three timepoints were analyzed by repeated-measures ANOVAs or Friedman test (repeated measure ANOVA on ranks). Αlpha was set at 0.05, Bonferroni and Tukey correction for multiple testing were applied if applicable. To test for multivariate effects of significant covariates of rTF_max_, we used stepwise multivariate regression models. In boxplots, horizontal lines represent mean values. Further, boxes quartiles and whiskers represent 5th and 95th percentiles. Scatterplots show individual data points unless indicated otherwise. All statistical plots were produced with Origin 2022.

## Results

### Baseline mental phenotype

The study sample had a mean age of 30.7 years (standard deviation (SD) 8.8, range 18–49) and was balanced in gender with 50.7% female and 49.3% male participants. For further demographics of the entire sample please see Table 1. Most participants (*N* = 44, 60.3%) of the study sample were diagnosed with MDD, followed by *N* = 23 inpatients (31.5%) suffering from BD and *N* = 6 inpatients (8.2%) suffering from SZD. With 84.7%, depressed episodes were dominating in the study sample, contrasting *N* = 2 participants (2.7%) with bipolar manic and *N* = 9 (8.2%) participants with bipolar mixed episodes. Due to low statistical power, these patient subgroups (bipolar manic, bipolar mixed) were excluded for further correlations between disease severity and rTF_max_. More than two thirds of the study sample (72.7%) stated their age of first disease onset with less than 30 years. One fifth (20.5%, *N* = 15) of the participants experienced their first depressive episode within the current treatment and 38.4% (*N* = 28) had not been hospitalized for an affective disorder before. At baseline, patients showed a mean MADRS score of 23.5 (SD 10.0), which equals a moderate depression. For further characterization please see Table 2.

**Table 1:** Demographic description of the study sample

Parameter	Reference		
Age [yrs]	18–50	30.74 ± 8.75 (18–49)	
		N	%
Gender	M/F	36/37	49.3/50.7
Marital status	Married	11	15.1
	Single	51	69.9
	Separated/divorced	7	9.6
	Widowed	1	1.4
	Missing	3	4.1
Education	Middle School	24	32.9
	A-level	8	11.0
	Apprenticeship/ training	27	37.0
	College/ university	11	15.1
	Missing	3	4.1
Employment status	Unemployed	23	31.5
	Retired	3	4.1
	Working part-time	9	12.3
	Working full-time	19	26.0
	Student/in training	16	21.9
	Missing	3	4.1
Net income per month	< 800 €	16	21.9
	801–1500 €	19	26.0
	1501–2000 €	13	17.8
	2001–3000 €	7	9.6
	3001–5000 €	3	4.1
	Missing	15	20.5

Data are reported as mean and standard deviation (SD). *Abbreviations f* female, *m* male, *N* sample number, *SD* standard deviation, *yrs* years

**Table 2:** Mental illness phenotype of the study sample

Parameter	Reference				
		N		%	
Main diagnosis	MDD	44		60.3	
	BD	23		31.5	
	SZD	6		8.2	
N of secondary psychiatric diagnoses	none	42		57.5	
	1	25		34.3	
	2	6		8.2	
Current episode of disease	depressed	61		84.7	
	manic	2		2.7	
	mixed	9		8.2	
Psychotic symptoms	yes	6		8.2	
	no	67		91.2	
Age of first onset [yrs]	< 18	26		35.6	
	18–29	27		37.1	
	30–39	20		13.7	
	40–49	4		5.5	
	not stated	6		8.2	
N of depressive episodes (incl. current)	1	15		20.5	
	2–3	8		10.9	
	4–5	13		17.8	
	> 5	20		27.5	
	not clearly definable	14		19.2	
	not stated	3		4.1	
N of previous inpatient stays	none	28		38.4	
	1–2	20		27.4	
	3–5	15		20.6	
	> 5	8		11.0	
	not stated	2		2.7	
Family History For affective disorder	y/n/missing	49/20/4		67.1/27.4/5.5	
History for suicide attempt(s)	y/n/missing	10/61/2		13.7/83.6/2.7	
**Psychometry**					
		t_1_	t_2_		t_3_
MADRS		23.48 ± 9.95, *N* = 73	16.66 ± 9.56, *N* = 67		16.61 ± 10.45, *N* = 62
YMRS		4.77 ± 6.20, *N* = 30	6.10 ± 6.61, *N* = 21		3.33 ± 3.58, *N* = 24
BPRS		47.0 ± 8.79, *N* = 7	37.75 ± 17.46, *N* = 8		37.43 ± 10.06, *N* = 7

Data are reported as mean and standard deviation (SD). Abbreviations: *BP* bipolar disorder, *BPRS* Brief Psychiatric Rating Scale, *MADRS* Montgomery–Åsberg Depression Rating Scale, *MDD* major depressive disorder, *N* sample number, *n* no, *SD* standard deviation, *SZD* schizo-affective disorder, *t* time point, *y* yes, *YMRS* Young Mania Rating Scale, *yrs* years

### Characterization of the patient’s physical status

With a mean BMI of 27.0 (SD 6.3) at baseline, participants were overweight and approximately equal to the mean BMI of German adults > 18 yrs (26.6 [60]), . Participants stated a mean sports activity [58, 59] of 2.5 h (SD = 4.4) per week before hospitalization. Sports frequency varied greatly with only *N* = 5 participants (6.8%) following a daily and *N* = 18 subjects (24.6%) following a every second day up to a weekly activity. This contrasted with 46.6% of the sample (*N* = 34) without regular sports frequency. Monitoring of possibly increased activity of CK and the lipid system (TG, TC, HDL, LDL) revealed no significant phenotype, further detailed in Table 3.

**Table 3:** Physical characterization of the study sample

Parameter	Reference			
		t_1_ (*N* = 73)	t_2_ (*N* = 67)	t_3_ (*N* = 62)
BMI [kg/m^2^]	≤ 25	27.03 ± 6.29	26.69 ± 5.74	27.08 ± 5.55
CK [U/l]		241.88 ± 1133.65^1^	131.15 ± 180.16	172.26 ± 245.29
TC [mg/dl]		173.38 ± 66.26	176.01 ± 62.98	170.21 ± 74.27
TG [mg/dl]		123.00 ± 97.91	125.96 ± 124.40	125.98 ± 81.81
HDL [mg/dl]		54.93 ± 61.60	48.87 ± 19.81	51.24 ± 21.61
LDL [mg/dl]		105.15 ± 41.57	105.76 ± 31.27	103.19 ± 32.97
Sports per week [h]		2.54 ± 4.44; *N* = 70		
			N	%
Sports frequency	Daily		5	6.8
	Every second day		9	12.3
	Once a week		9	12.3
	Several times a month		13	17.8
	Not so often		34	46.6
	Not stated		3	4.1
N of somatic diseases	none		27	37.0
	1–2		36	49.3
	3–4		10	13.7
Smoking	y/n/missing		27/43/3	37.0/58.9/4.1
Suspected alcohol related disorder	y/n/missing		8/62/3	11.0/84.9/4.1

Data are reported as mean and standard deviation (SD). The suspected alcohol related disorder was defined by an AUDIT score ≥ 8. Abbreviations: *AUDIT* alcohol use disorders identification test, *BMI* body mass index, *CK* creatin kinase, *HDL* high density lipoprotein, *LDL* low density lipoprotein, *N* sample number, *n* no, *t* time point, *TC* total cholesterol, *TG* triglycerides, *y* yes, *yrs* years

^1^One patient displayed a baseline CK value of 9769 U/I which was controlled in repeated measurements

### Measurement of maximal muscle strength in a clinical setting

As illustrated in Fig. 1, the study sample underwent a strength circuit which enabled the observational measurement of the maximal muscle strength of four different body regions at three timepoints (upper extremity, lower extremity, back, and abdomen, Fig. 3A). At t_1_, muscle strength of the lower extremity was the major contribution (51.8%) to rTF_max_ in the patient sample (Fig. 3A). Male and female participants significantly differed in their mean rTF_max_ (6.41 (f, *N* = 35) vs. 10.50 (m, *N* = 35), *p* < .001) resulting in gender as a covariate for subsequent calculations.

### Association between maximal muscle strength and disease severity and history

Comparing all time points in the entire depressed study sample, the mean MADRS score showed a significant decrease (*p* < .001, repeated measures ANOVA), while the median rTF_max_ significantly increased over all time points (*p* < .001, Friedman test, Fig. 3B). After correction for multiple testing, this change for both parameters was significant between t_1_ and t_2_ (*p* < .001) but not t_2_ and t_3_ (*p* > .05, Bonferroni and Tukey, respectively, Fig. 3B). To evaluate the temporal change of rTF_max_, we controlled for BMI changes over time with no significant difference in the study sample and between genders (Table 3). As previously described, we assumed an interindividual association between depression severity and rTF_max_ in the depressed study sample (*N* = 61, Table 4), but found no significant correlations in the sample and between genders at any of the time points (*p* > .05). To account for intraindividual associations of both parameters, we calculated and correlated the differences (Δ) of the MADRS and rTF_max_ scores with no significant outcome at all three time points (*p* > .05). Lastly, ΔrTF_max_ was calculated in therapy responders (≥ 50% reduction of baseline MADRS [34]), and therapy remitters (MADRS ≤ 12 [34]), vs. non-responders and non-remitters with regards to the depressive symptoms. For therapy responders, the mean rTF_max_ showed an increasing trend compared to non-responders (*p* = .054), while therapy remitters vs. non-remitters showed no significant differences (*p* = .356).

**Table 4:** Bivariate Pearson correlation between maximal muscle strength mental and somatic covariates at time point 1

Parameter	Study Sample			Female Sample			Male Sample		
	N	R	p	N	r	p	N	r	p
*Mental*									
Disease severity (MADRS, t_1_)	59	-0.105	0.430	30	-0.214	0.255	29	0.212	0.270
*Psychopharmacology*									
rD_AM_	69	0.124	0.308	35	0.104	0.554	34	0.013	0.940
rD_IM_	69	-0.258	0.032*	35	-0.477	0.004	34	-0.347	0.044*
rDT	69	0.250	0.038*	35	0.400	0.017*	34	0.217	0.217
*Disease history*									
duration	55	-0.068	0.622	29	-0.406	0.029	26	0.072	0.728
Number of episodes	47	0.031	0.834	25	0.006	0.976	22	0.126	0.545
Disease load	47	0.172	0.249	25	0.331	0.106	22	0.196	0.381
Number of previous inpatient stays	58	-0.261	0.048*	30	-0.400	0.028*	28	-0.304	0.115
Number of secondary mental diagnoses	59	0.205	0.120	30	0.386	0.035*	29	-0.041	0.834
TICS	68	-0.02	0.873	34	0.135	0.448	34	-0.006	0.973
*Physical health*									
Gender	70	0.652	< 0.001***						
Age	70	-0.292	0.014*	35	-0.449	0.007**	35	-0.177	0.309
Sports per week	68	0.105	0.395	34	0.354	0.040*	34	0.134	0.451
Number of somatic comorbidities	70	-0.011	0.931	35	-0.079	0.651	35	-0.250	0.147
Smoking	67	0.236	0.056	34	0.247	0.159	34	-0.211	0.231
Suspected alcohol related disorder	67	0.160	0.195	34	0.169	0.339	34	0.009	0.959
*Lipid system*									
TC	70	0.027	0.822	35	-0.171	0.326	35	0.109	0.534
TG	70	-0.141	0.244	35	-0.409	0.015*	35	-0.252	0.144
HDL	70	-0.101	0.404	35	-0.013	0.939	35	0.443	0.008**
LDL	70	-0.093	0.445	35	-0.380	0.024	35	-0.067	0.701

Asterisks in the table indicate the level of significance (∗*p* < .05, ∗∗*p* < .01, ∗∗∗*p* < .001). Abbreviations: *HDL* high density lipoprotein, *LDL* low density lipoprotein, *N* sample number, *N* sample number, *p* probability value, *r* Pearson’s correlation coefficient, *rD*_*AM*_ relative dosage of activating medication, *rD*_*IM*_ relative dosage of inhibiting medication, *rDT* relative dosage of the total (AM + IM) medication, *t* time point, *TC* total cholesterol, *TG* triglycerides, *TICS* Trier Inventory of Chronic Stress

Based on these unexpected findings, we evaluated other parameters of the patient’s mental illness phenotype. We tested for an association of previous disease history and rTF_max_. Interestingly, the number of previous hospitalizations inversely correlated with rTF_max_ (total: *r* = − .261, *p* = .048; f: *r* = − .400, *p* = .028; m: *r* = − .344, *p* = .050); females further showed a negative correlation with disease history (*r* = − .406, *p* = .029) and number of secondary mental disorder diagnoses (*r* = .386, *p* = .035). Thus, while we could not elicit a direct correlation of the MADRS score and rTF_max_, disease history was shown to be relevant for rTF_max_.

### Association between maximal muscle strength and psychopharmacotherapy

Based on the assumption that psychopharmacotherapy influences rTF_max_, we introduced an approximation model combining a classification by Benkert and Hippius [55] in activating and inhibiting medication (AM and IM, see Supplementary Table 1) and the normalizing parameter of the defined daily dose (DDD [56],see Methods). Relative doses (rD) of each medication were added separately in the two groups of AM and IM.

**Fig. 4:**
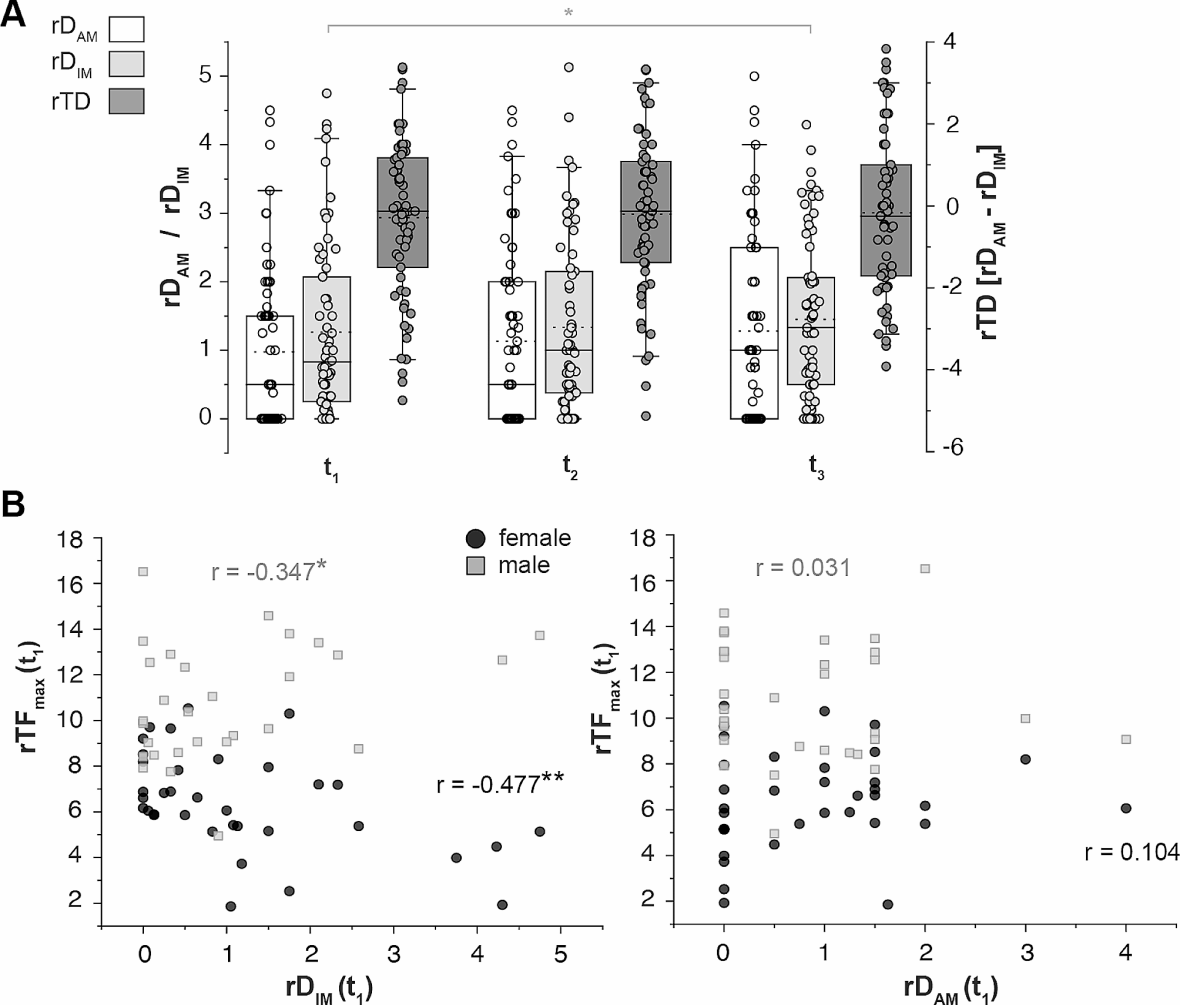
Maximal muscle strength correlates with inhibiting but not activating medication. **A**: Summary graphs of the relative dosage of activating (rD_AM_, white box) and inhibiting medication (rD_IM_, light grey, *p* = .043, after Tukey correction, Friedman test) as well as the difference of both relative dosages, the total relative dosage (rTD, dark grey), during the study course (*N* = 61). **B**: Summary graphs of relative total maximal muscle strength ( rTF_max_) and the relative dosage of the inhibiting medication (rD_IM_, left panel) as well as activating medication (rD_AM_, right panel) at t_1_. Pearson’s correlation coefficient r was calculated. Horizontal lines in boxplots represent median, dotted horizontal lines represent mean; boxes 25th and 75th quartiles; whiskers 5th and 95th percentiles; scatter plots show individual data points. Asterisks indicate significance level (*p < .05, **p < .01, ***p < .001)

First, we evaluated the temporal change of AM and IM over all time points in the entire study sample, including the bipolar patients. Both the median relative dosage of activating substances (rD_AM_) and inhibiting medication (rD_IM_) increased over time (*N* = 61, *p* = .006 (rD_AM_), *p* = .017 (rD_IM_), Friedman test, Fig. 4A). After multiple comparison corrections, rD_AM_ did not significantly differ between the time points, while rD_IM_ significantly increased with 160% between t_1_ and t_3_ (*p* = .043). As patients received both substance classes simultaneously and to account for intra-individual changes, we controlled for the median difference between rD_AM_ and rD_IM_, termed as the total relative dosage (ΔrTD), indicating a more activating or inhibiting change of medication over time (see Methods). There was no significant change found at any of the time points (*p* = .453, Fig. 4A).

Next, we tested for correlations between rTF_max_ and psychopharmacotherapy. We found that rD_IM_ significantly correlated with a lower rTF_max_ at t_1_ (*r* = − .258, *p* = .032, Fig. 4B). This was also aggravated in females, even at t_2_, compared to males (female: t_1_, *r* = − .477, *p* = .004; t_2_, *r* = − .358, *p* = .044 vs. male: t_1_, *r* = − .347, *p* = .044). This correlation was not observed for rD_AM_ (Fig. 4B). Only for the female cohort, ΔrTD positively correlated with rTF_max_ between t_2_ and t_3_ (*r* = .422, *p* = .035, *N* = 25). While we found no significant correlation for depression severity, rD_IM_ was significantly associated with longer disease duration (effect size between *r* = .253 and 0.465, *p* < .05) and number of hospitalizations (effect size between *r* = .342 and 0.716, *p* < .05). Thus, we tested for differences in the MADRS items 3 (inner tension), 4 (reduced sleep), and 9 (pessimistic thoughts), which might account for higher dosages of IM, in more severely ill patients (≥ 3 hospitalizations; median 7.83, SD 3.16, *N* = 23) vs. the rest of the study sample (median 8.27, SD 3.21, *N* = 48) without any significance.

### Patient’s physical health influences maximal muscle strength

As the ability for PA is highly determined by the patient’s basic physical health, we evaluated somatic covariates of the study sample at baseline. An increased age was significantly associated with decreased rTF_max_ (*r* = − .292, *p* = .014 (total sample), *r* = − .449, *p* = .007 (f), *r* = .122, *p* = .307 (m)). Using a point-biserial correlation, tobacco smoking and a suspected alcohol related disorder (defined by an AUDIT score ≥ 8 [38]), had no significant correlation with rTF_max_. Secondly, the TICS score indicating a chronic stress level was not significantly correlated with rTF_max_. For females, we found a significant correlation of the weekly sports activity before admission (*r* = .354, *p* = .040). Regarding the lipid profile, TG and LDL scores inversely correlated with rTF_max_ in females (*r* = − .409, *p* = .015 and *r* = − .414, *p* = .023), whereas in males higher HDL scores were significantly associated with higher rTF_max_ (*r* = .443, *p* = .008). Please see Table 4 for further details.

Lastly, we evaluated a possible influence of the participation in the sports therapy program of the clinic which generally intended to offer supervised PA in a stationary inpatient setting with reduced possibilities for any kind of daily activity (see Methods and Supplementary Table 2 for a detailed description). *N* = 60 (89.6%) of *N* = 67 patients participated in the sports therapy program between t_1_ and t_2_, *N* = 46 (*N* = 73.0%) of *N* = 63 patients between t_2_ and t_3_. However, we found no significant correlation of the participation in a sports group and rTF_max_. in any of the investigated groups (*p* > .05).

### Gender, inhibiting medication, and age determining maximal muscle strength

Based on our bivariate analyses, we integrated gender, rD_IM_, and age as significant covariates on rTF_max_. Our stepwise regression model revealed that these three covariates account for the majority of variance in rTF_max_ (corrected R^2^ = 0.584, F = 25.353, *p* < .001; *N* = 53). Differentiating between gender, rD_IM_ and age significantly influenced rTF_max_ in females (corrected R^2^ = 0.373, F = 8.731, *p* = .001; *N* = 27), while only the HDL score was a significant factor in males (corrected R^2^ = 0.169, F = 6.091, *p* = .021; *N* = 26).

## Discussion

### No correlation between depression severity and maximal muscle strength

We found no correlation between depression severity (MADRS) and rTF_max_ in our study sample (Table 4; Fig. 3B). A recent large cohort study [61] and meta-analysis [24]) revealed that muscle strength and muscular fitness are inversely associated with a higher incidence of depression and with depressive symptoms in middle-aged to older people. A few studies even suggest a significant association independent of the level of PA [24, 62]. Here, we introduce an investigation in a comparably young and sportive patient sample (mean age 30.7 yrs, Table 1), which matched the WHO recommendations of 150–300 min for adults with a mean activity of 2.5 h per week [63]), but also presented with a certain motivation to participate in this comprehensive study paradigm in a clinical setting (selection bias). Thus, several reasons might account for our contradictory findings: (i) Young inpatients with less comorbidities and shorter disease history might be more likely to outweigh (psycho-)motor alterations. This is supported by the significant inverse correlations of age and disease history but also the correlations of the patients’ physical status on rTF_max_ in our study sample (Table 4). (ii) The study duration of four weeks was strategically designed based on the assumption that muscle mass would not significantly change within this timeframe [49, 64]. Our observed significant increase of rTF_max_ in the first two weeks and the following plateau of rTF_max_ (Fig. 3B) might account for fast neuromuscular adaptions rather than muscle mass development which occurs over several weeks [49, 64]. In parallel, the low remission rate of 34% in our study sample underpins that patients were still moderately to severely depressed. Interestingly, a recent Chinese cohort study with of over 13,000 middle-aged to older patients and four years follow up matches with our findings [65]. They revealed a non-linear L-shaped association of grip strength as parameter of muscle strength [66] and depressive symptoms (see Fig. 2 in [65]). Translated to our present study, muscle strength would reach a declined plateau (see t_2_ and t_3_, Fig. 3B), once the patient is moderately to severely depressed. (iii) In contrast to using (hand) grip strength as parameter, we decided for fitness circuit devices mirroring complex movements and PA in the patient’s daily life. These were successfully implemented in earlier studies (e.g [46, 47]. however, with our total score more focussing on the lower extremity muscle strength (see Fig. 3A).

### Inhibiting but not activating medication influences maximal muscle strength

The relative dosage of inhibiting medication was inversely correlated with rTF_max_, particularly in women. This finding is supported by a recent review by Hirschbeck et al. [11], in which antipsychotics (APs) were found to have primarily impairing effects on physical performance. APs were received by 62.5% of our study sample. Despite the broad evidence in schizophrenic patients that this drug class impacts the motor system (e.g. for overview [67]), our results highlight possible (daily) motoric impairments for patients suffering from affective disorders and possibilities for preventive measurements, especially in young and female patients.

No significant positive correlation of activating substances, including stimulants and reuptake inhibitors, on muscle strength could be detected. In recent studies, stimulant medication is discussed to enhance physical performance [68–70]. However, different classes of reuptake inhibitors are evaluated more contradictory [11] which may underpin our findings in a cohort of mainly depressed inpatients. Further, most studies investigate effects in healthy athletic subjects than (severely) ill inpatients.

#### Limitations of the study

Beside the above-mentioned, our study design protocol faces the naturalistic and observational inpatient setting as foremost limitation which hampers a randomized controlled study design with matched psychopharmacotherapy and healthy controls. The presence of unavoidable polypharmacy (Fig. 4A) in severely ill patients may further shadow effects of specific medication classes on muscle strength. Besides, larger sample numbers are needed to investigate a correlation between disease severity of illness in bipolar manic/mixed and schizoaffective manic/mixed inpatients and muscle strength in depth. Additionally, improved conditions of the strength measurement procedure, such as standardization in daytime and verbal instructions, follow-up, e.g. at eight to twelve weeks’ time, and healthy control measurements as well as a specific test-rest-reliability experiment for the used protocol would be beneficial for further interpretation of the obtained strength values. While there was no significant correlation of participation in the clinical sports therapy program and rTF_max_, it would be of interest to investigate selected sports therapy interventions in a randomized controlled study design in future studies.

### Future implications

To delineate the dynamics of mood and muscle strength in a more native context, ecological momentary assessments (EMAs [71]), widely used in psychology and psychiatry, appear to be a promising approach, especially for a digitally oriented young inpatient sample. By repeated sampling of the patients’ current mood and muscle strength in real time, e.g. via surveys on smartphones and physiological sensors, EMAs may help to assess possible interaction between both parameters more sensitively than the current study design. Integrating these EMAs into a deep phenotyping approach of depressed inpatients (e.g [72]), including therapeutic drug monitoring [73]), may help to personalize (drug) treatment, to monitor motor alterations and to foster PA in young severely ill MDD, BP and SZD patients.

## Conclusion

Here, we introduce a prospective observational study which intended to investigate the relationship of muscle strength, mood, and moderating effects of psychopharmacotherapy in a sample of young to middle-aged inpatients diagnosed with affective disorders. We combined a strength circuit as observational measurement with psychometry and an approximation model of medication in a clinical setting. While a bivariate correlation between depression severity and muscle strength was not detected in the present study sample, inhibiting psychopharmaceuticals correlated significantly with reduced muscle strength.

Based on regression models indicating a substantial effect of medication, gender, and age on muscle strength, future research will be needed to develop measurements for prevention of muscle weakness, especially in younger (in-)patients with chronic affective illnesses.

### Electronic supplementary material

Below is the link to the electronic supplementary material.

Supplementary Material 1; Supplementary Table 1. Activating and inhibiting psychopharmaceuticals according to Benkert & Hippius 2021(Benkert & Hippius, 2021).

## Data Availability

The datasets used and/or analysed during the current study are available from the corresponding author on reasonable request.

## Abbreviations

AM: Activating medication
AUDIT: Alcohol use disorders identification test
BD: Bipolar affective disorder
BPRS: Brief Psychiatric Rating Scale
CK: Creatine kinase
DDD: Defined daily dose
F_max_: Maximal muscle strength
HDL: High density lipoprotein
ICD-10: Statistical Classification of Diseases and Related Health Problems Version 10
IM: Inhibiting medication
LDL: Low density lipoprotein
MADRS: Montgomery Ǻsberg Depression Rating Scale
MDD: Major depression disorder
N: Sample number
n: no
p: Probability value
PA: Physical activity
PTSD: Post-traumatic stress disorder
r: Pearson’s correlation coefficient
rD: relative dose
rD_AM_: rD of activating medication
rD_IM_: rD of inhibiting medication
rDT: rD of the total (AM + IM) medication
rTF_max_: relative total F_max_
SD: Standard deviation
SF-36: Short-form-36 health survey
SZD: Schizo-affective disorder
t_1_-t_3_: Time points
TC: Total cholesterol
TG: Triglycerides
TICS: Trier Inventory for Chronic Stress
y: yes
TICS: Trier Inventory for Chronic Stress
YMRS: Young mania rating scale
yrs: years
