# Supplementary material for: The association between maximal muscle strength, disease severity and psychopharmacotherapy among young to middle-aged inpatients with affective disorders – a prospective pilot study
Source: BMC Psychiatry. 2024 May 29;24:401. doi: 10.1186/s12888-024-05849-2 (PMC11137909; doi:10.1186/s12888-024-05849-2)
Supplement: Supplementary file 1 — Supplementary Material 1; Supplementary Table 1. Activating and inhibiting psychopharmaceuticals according to Benkert & Hippius 2021(Benkert & Hippius, 2021). [file 12888_2024_5849_MOESM1_ESM.docx]

**Supplement**

**Supplementary Table 1. Activating and inhibiting psychopharmaceuticals according to Benkert & Hippius 2021 (Benkert & Hippius, 2021).**

| **activating medication (AM)** | | **inhibiting medication (IM)** | |
| --- | --- | --- | --- |
| antidepressant medication | | antidepressant medication | |
|  | SSRI |  | TCA |
|  | SNRI |  | NaSSA |
|  | NDRI |  | SARI |
| ADHD medication | |  | MRA |
|  | stimulants |  | esketamine |
|  | non-stimulants | anxiolytic medication | |
|  | |  | BZD |
|  |  | mood stabilizers | |
|  |  |  | anticonvulsants |
|  |  |  | lithium |
|  |  | antipsychotic medication | |
|  |  | antihistaminic medication | |

Abbreviations: *ADHD* attention deficit hyperactivity disorder*, BZD* benzodiazepines*, MRA* melatonin receptor agonists*, NaSSA* noradrenergic and specific serotonergic antidepressants*, NDRI* norepinephrine-dopamine reuptake inhibitors*, SARI* serotonin antagonist and reuptake inhibitors*, SNRI* serotonin-norepinephrine reuptake inhibitors*, SSRI* selective serotonin reuptake inhibitors, *TCA* tricyclic antidepressants.

**Supplementary Table 2. Overview of sports therapy groups at the Department of Psychiatry, Psychosomatics and Psychotherapy, University Hospital Würzburg, during the study.**

**.**

| **group type** | | **procedure** | **duration (h)** |
| --- | --- | --- | --- |
| higher intensity | |  |  |
|  | morning sports group ‘FIT’ | exercise with body weight or other weights (squat, circuit training) | 0.5 |
|  | nordic walking | walking in mountainous areas (ascending and descending trails) | 1 |
|  | activation group | open field games, e.g. volleyball, basketball, badminton, frisbee | 1 |
| lower intensity | |  |  |
|  | morning sports group ‘EASY’ | activating exercises (stretching, exercises while sitting) | 0.5 |
|  | fitness group 1 | open field games, e.g. croquet, boccie | 1 |
